# Dissociation of reward and effort sensitivity in methcathinone‐induced Parkinsonism

**DOI:** 10.1111/jnp.12122

**Published:** 2017-04-05

**Authors:** Trevor T.‐J. Chong, Valerie Bonnelle, Kai‐Riin Veromann, Julius Juurmaa, Pille Taba, Olivia Plant, Masud Husain

**Affiliations:** ^1^ Monash Institute of Cognitive and Clinical Neurosciences Monash University Clayton Victoria Australia; ^2^ Department of Experimental Psychology University of Oxford UK; ^3^ Nuffield Department of Clinical Neurosciences University of Oxford UK; ^4^ Department of Neurology and Neurosurgery University of Tartu Estonia

**Keywords:** Parkinsonism, methcathinone, motivation, effort, reward

## Abstract

Methcathinone‐induced Parkinsonism is a recently described extrapyramidal syndrome characterized by globus pallidus and substantia nigra lesions, which provides a unique model of basal ganglia dysfunction. We assessed motivated behaviour in this condition using a novel cost‐benefit decision‐making task, in which participants decided whether it was worth investing effort for reward. Patients showed a dissociation between reward and effort sensitivity, such that pallidonigral complex dysfunction caused them to become less sensitive to rewards, while normal sensitivity to effort costs was maintained.

## Background

Patients with basal ganglia pathology, including those with idiopathic Parkinson's disease, can show significant deficits in motivation, independent of their motor impairment (Chong *et al*., 2015). However, the mechanisms underlying such motivational deficits are poorly understood. Motivation requires us to weigh the costs of an action against its potential benefits (Salamone & Correa, 2012). Individuals have to assess not only the amount of reward necessary to motivate an action – their *reward sensitivity* – but also how much effort they are willing to exert for a potential reward–*effort sensitivity*.

Although animal studies suggest that the mesolimbic pathway from the ventral tegmental area (VTA) to the ventral striatum is critical to such cost‐benefit valuations, far less is known of the role of the globus pallidus (GP) and substantia nigra (SN), which are primary recipients of ventral striatal projections (Haber, Lynd, Klein, & Groenewegen, 1990). Rare case studies have reported motivational impairments such as apathy following GP lesions (Adam *et al*., 2013). However, the mechanisms underlying this effect and, in particular the functional role of the pallidonigral complex in differentially modulating reward and effort sensitivity, have not been explored.

Recent reports of a distinctive syndrome of manganism amongst intravenous methcathinone users in Eastern Europe offer a unique opportunity to address this issue (Stepens *et al*., 2008). Methcathinone is a euphoric stimulant manufactured by oxidising ephedrine and pseudoephedrine with potassium permanganate. Manganese has a particular affinity for the basal ganglia, in particular the GP and SN (Redgrave *et al*., 2010). The neurotoxic effects of manganese in these regions results in a characteristic, extrapyramidal syndrome of symmetrical hypokinesia, dystonia, postural instability and dysarthria. Given its distinctive pathophysiology, this syndrome may offer a rare insight into the functional role of the pallidonigral complex to motivated behaviour.

Here, we tested patients with methcathinone‐induced Parkinsonism on a novel cost‐benefit decision‐making task (Chong *et al*., 2015). On a trial‐by‐trial basis, participants decided whether to accept or reject a potential reward based on the effort required to obtain it. We then determined, for each effort level, the reward required to motivate participants to accept an offer – their *reward indifference points*. Similarly, we calculated, for each reward, the amount of effort that individuals were willing to invest towards it – their *effort indifference points*. We then used these measures to compare reward and effort sensitivities between patients and controls.

## Methods

### Participants

**Figure 1:**
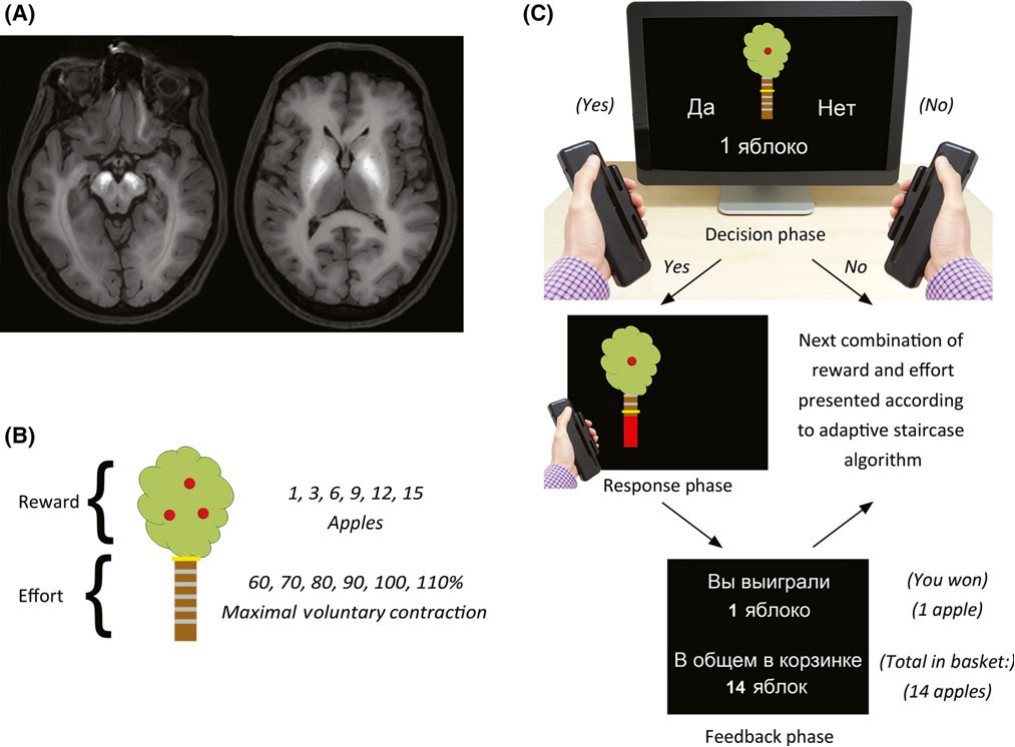
The apple gathering task. (A) Axial slices from a representative patient showing lesions in the substantia nigra (left) and globus pallidus (right). (B) Rewards were indicated by the number of apples on the tree, while the associated effort was indicated by the height of a yellow bar positioned at one of six levels on the tree trunk. (C) On each trial, participants decided whether they were willing to exert the specified level of effort for the specified reward. If they judged the particular combination of reward and effort to be ‘not worth it,’ they selected the ‘No’ response. If, however, they decided to engage in that trial, they selected the ‘Yes’ response, and then had to squeeze a hand‐held dynamometer with a force sufficient to reach the target effort level. Participants received visual feedback of their performance, as indicated by the height of a red force feedback bar. To reduce the effect of fatigue, participants were only required to squeeze the dynamometers on 50% of accepted trials. At the conclusion of each trial, participants were provided with feedback on the number of apples gathered. [Colour figure can be viewed at http://www.wileyonlinelibrary.com]

Seven Russian‐speaking Estonian cases and 18 matched controls were tested (Table 1). Patients had a confirmed diagnosis by a neurologist of Parkinsonism secondary to methcathinone. The average duration of methcathinone use was 3.5 years, and all patients denied ongoing use. Those who were scanned acutely (3 of 7) had confirmed pallidonigral lesions, without other abnormalities (Figure 1A; Table S1). The remaining four were not seen until after cessation of drug use and did not show evidence of active lesions, consistent with previous longitudinal observations which report resolution of MR changes despite persistent extrapyramidal signs (Stepens *et al*., 2008). Controls were free of neurological illness, and denied current or previous drug use.

**Table 1:** Summary of participant demographics (means ± *SE*)

	Patients (*n *=* *7)	Controls (*n *=* *18)	Difference
Gender (M:F)	6:1	11:7	χ^2^(1, 25) = 1.40, *n.s*.
Age	39.1 (1.0)	35.9 (2.6)	*t*(23) = 0.93, *n.s*.
Education	11.1 (1.5)	11.1 (0.7)	*t*(23) = 0.10, *n.s*.
UPDRS	69.7 (9.53)	N/A	N/A
Duration of methcathinone use	5.1 years (2.2)	N/A	N/A
LARS	−16 (2.7)	−25 (1.6)	*t*(23) = 2.57, *p *=* *.02
BDI	20.7 (5.7)	8.1 (1.1)	*t*(23) = 1.91, *n.s*.

### Methods

The paradigm was identical to that of a previous study (Chong *et al*., 2015), but administered in Russian. Participants were seated in front of a computer interfaced with hand‐held dynamometers. At the beginning of each session, the dynamometers were calibrated to each participant's maximal voluntary contraction (MVC).

On each trial, participants were presented with an image of an apple tree, and instructed to accumulate as many apples over the experiment (Figure 1B,C). The maximum potential reward was indicated by the number of apples on the tree (1, 3, 6, 9, 12, 15), while the effort required to obtain that reward was indicated by the height of a yellow bar on the trunk, which varied as a function of each participant's MVC (60–110%). By referencing the effort levels to each individual's maximum force, we normalized the difficulty of each level across individuals. A different combination of reward and effort was presented on each trial.

Importantly, this task measured participants’ *decisions* to perform an action, rather than the effort manifest in the actions themselves. On each trial, participants decided whether they were willing to exert the specified level of effort for the specified reward. If they judged the particular combination of reward and effort to be ‘Not worth it,’ they selected the NO response, and the next trial commenced. If, however, they decided to engage in that trial, they selected the YES option, and had five‐seconds to squeeze the dynamometer to reach the target level.

## Results

### Reward indifference points

**Figure 2:**
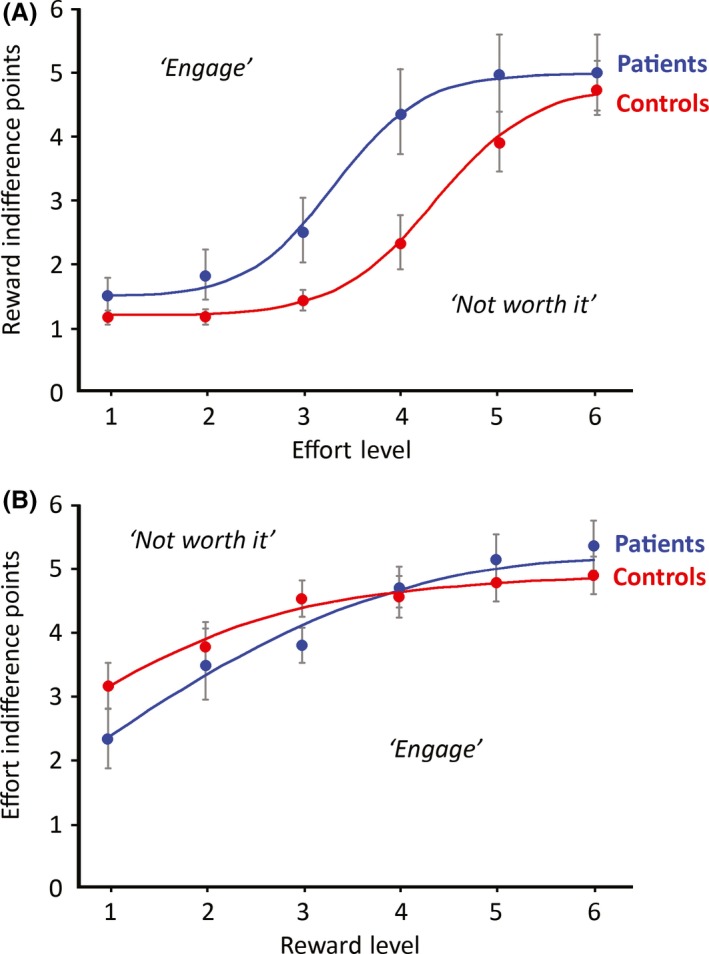
Patients in the effort–reward decision‐making task showed a dissociation between reward and effort sensitivity. (A) Reward indifference points refer to the reward for which the probability of engaging in a trial at a given effort level is 50%. Reward indifference points divide the reward–effort space into a sector in which participants are willing to engage in an effortful response (above the curve) from a sector that is judged ‘not worth it’ (below the curve). Patients needed to be incentivized with greater reward to invest effort. Error bars indicate ± 1 *SEM*. (B) The analogous plot for effort indifference points, which showed no difference between patients and controls. [Colour figure can be viewed at http://www.wileyonlinelibrary.com]

How much reward was required to motivate participants to exert each level of effort? For each *effort level*, we estimated the reward at which the probability of accepting an offer was 50% – the *reward indifference points*. For each participant, we fitted a logistic function to the choice probability data for each reward. The reward indifference points thus derived were then plotted against the corresponding effort levels (Figure 2A). Reward indifference points for patients and controls were compared using a repeated‐measures ANOVA, with the between‐subjects factor of Group (patients, controls) and the within‐subject factor of Effort (Levels 1–6).

Importantly, the main effect of Group was significant, such that the reward indifference points were higher for patients than controls, *F*(1, 23) = 4.6, *p *<* *.05, $\eta_{p}^{2}$ = .17. The main effect of Effort was also significant, showing that reward indifference points increased with effort, *F*(2.0, 45.4) = 35.9, *p *<* *.001, $\eta_{p}^{2}$ = .61; Greenhouse‐Geisser corrected, but the Group × Effort interaction was not significant, *F*(2.0, 45.4) = 1.71. These results indicate a *lower reward sensitivity* relative to controls.

### Effort indifference points

Next, we asked how much effort people were willing to exert for different levels of reward (Figure 2B). For each reward, the *effort indifference point* was defined as the probability of accepting an offer 50% of the time. An analogous Group × Reward ANOVA revealed a main effect of Reward, *F*(2.2, 51.6) = 23.4, *p *<* *.001, $\eta_{p}^{2}$ = .51, such that effort indifference points increased with reward, as predicted. However, there was neither a significant effect of Group, *F*(1, 23) = 0.77, nor a Group × Reward interaction, *F*(2.2, 51.6) = 2.13. This demonstrates that patients were no less willing than controls to invest effort for each level of reward, and indicates identical effort sensitivities across groups.

### Maximum voluntary contraction

Lastly, to ensure that the differences in reward sensitivity were not attributable to differences in force output, we compared MVCs in the two groups. Importantly, they were not significantly different, *t*(23) = 0.16. In addition, we examined the effect of fatigue on motor performance by comparing changes in maximum force output over the course of the test, but this was no different between the two groups, *t*(23) = 0.98. Thus, the higher reward indifference points in patients were not simply due to a reduced capacity to exert force.

## Discussion

Patients with methcathinone‐induced Parkinsonism had significantly reduced sensitivity to reward compared to matched controls, but their sensitivity to effort costs remained unchanged. Importantly, these deficits in reward sensitivity were manifest in *choice behaviour*, even prior to the execution of the action itself. Furthermore, they were independent of any motor deficits, as there were no significant differences in force (MVC) between the two groups. Together, the dissociation between reward and effort sensitivity in patients with methcathinone‐induced parkinsonism suggests that the pallidonigral complex plays a critical role in biasing computations towards potential rewards relative to effort costs.

Motivated decision‐making involves a complex network of brain areas. The connections between the basal ganglia and medial prefrontal areas are considered crucial to reward valuation (Walton, Rudebeck, Bannerman, & Rushworth, 2007), and the GP and SN play important roles in relaying mesolimbic output to higher cortical areas (Haber *et al*., 1990). Our results indicate that the functional consequence of this pallidonigral degeneration might be to impair reward‐based decisions, suggesting a key role for this complex in relaying information related to *reward sensitivity*.

Interestingly, however, *effort sensitivity* was preserved in patients versus controls. This suggests the presence of relatively intact pathways tuned to effort relative to reward‐based computations. Although the principal projections from the striatum are to the pallidonigral complex, the basal ganglia may also influence cortical areas involved in cost‐benefit valuations through alternative routes. For example, the mesolimbic pathway projects to non‐basal ganglia nuclei, and the VTA contains direct projections to prefrontal cortical areas via the mesocortical route (Bentivoglio & Morelli, 2005).

The dissociation between reward and effort sensitivity therefore suggests a division of labour between a reward‐sensitive pathway through the pallidonigral complex, and an effort‐sensitive pathway that bypasses it. Interestingly, midbrain dopaminergic synthesis is relatively preserved in methcathinone‐induced parkinsonism (Guilarte, 2013), which provides a potential mechanism by which the dopaminergic midbrain may continue to exert a direct influence on prefrontal areas. This contrasts with the pathophysiology of idiopathic Parkinson's disease, in which reduced midbrain dopamine synthesis likely accounts for the reduced effort sensitivity in that population (Chong *et al*., 2015; Le Bouc *et al*., 2016).

In summary, patients with methcathinone‐induced Parkinsonism appear to have selective impairments in reward sensitivity in the presence of intact effort sensitivity. This demonstrates the dissociability of reward and effort sensitivity, which may each be driven by different neuroanatomical substrates. More broadly, this implies that diseases affecting different components of the basal ganglia may result in distinct amotivational syndromes, elements of which may be dissected using paradigms that are sufficiently sensitive to these differences.

## Supporting information


**Data S1.** Methodological and analytical details, together with further discussion.
