# Supplementary material for: Dissociation of reward and effort sensitivity in methcathinone‐induced Parkinsonism
Source: J Neuropsychol. 2017 Apr 5;12(2):291–7. doi: 10.1111/jnp.12122 (PMC6001457; doi:10.1111/jnp.12122)
Supplement: Supplementary file 1 — Data S1. Methodological and analytical details, together with further discussion. [file JNP-12-291-s001.docx]

**Supporting Information**

1. **Supplementary Method**
   1. *Participant Recruitment*

Patients were recruited from a clinical database of methcathinone abusers. The criterion for inclusion was a diagnosis of methcathinone-induced parkinsonism, which implied the presence of a clinical syndrome of parkinsonism and dystonias on a background of home-made methcathinone mixture abuse. This diagnosis was made by a specialist neurologist (PT). Healthy age-matched controls were recruited from the general community. Patients were excluded if they were physically unable to perform or understand the task requirements (one patient). As in the original version of the task, participants were not paid for their participation (Chong et al., 2015).

- 1. *Calibration Phase*

At the beginning of each session, the dynamometers were calibrated to each participant’s maximal voluntary contraction (MVC). Participants alternately squeezed the left and right dynamometers as strongly as possible, while receiving real-time feedback on their contraction. Following the first trial, participants were instructed to squeeze each dynamometer on two additional trials to reach a target horizontal line. Importantly, this target line was deliberately set at 110% of their previous maximum contractions (i.e., which should be outside their capacity to perform). The purpose of this target line was two-fold: 1) To incentivise participants to squeeze to their maximum capacity; and 2) To flag participants whose MVCs were poorly calibrated. Any individuals who were able to exceed the target force of 110% MVC were recalibrated.

- 1. *Familiarisation Phase*

Prior to commencing the main task, participants were familiarised with the amount of force required to achieve each effort level. During these preliminary trials, images of trees without apples were presented. They were told that the height of the horizontal bar on the tree trunk was proportional to the amount of force they would need to exert in the main experiment. However, they were not explicitly informed about the percentage MVC corresponding to each level. In this preliminary phase, participants had the opportunity to familiarise themselves with the amount of force required for each effort level by squeezing the dynamometers on separate trials to attempt to achieve each target effort level (two familiarisation trials per effort level).

1. **Supplementary Analyses**
   1. *Correlation between LARS and Indifference Points*

Patients with methcathinone-induced parkinsonism were significantly more clinically apathetic than control participants when assessed on the Lille Apathy Rating Scale (LARS) (-16 ± 2.7 vs -25 ± 1.6, *t*(23) = 2.57, *p* < .05). This is consistent with reports of apathy being commonly reported in individuals with methcathinone-induced parkinsonism (Poniatowska et al., 2014; Selikhova et al., 2016), and more generally in cases of manganese neurotoxicity (Rodier, 1955).

We tested whether LARS scores predicted effort and reward sensitivity after collapsing data across all participants (patients and controls). We performed a robust regression with Huber correction, using LARS scores as the predictor variable, against mean effort or reward indifference points. Across the entire sample of 25 individuals, LARS scores reliably predicted the reward indifference points (β = 0.06; *p* < .001).This result is directionally consistent, such that higher scores on the LARS (i.e., greater apathy) significantly predicted the need to incentivise individuals with higher rewards. Interestingly, however, LARS scores did not predict the effort indifference points (β = -0.04; *p* = .14).

Together, these results suggest that the LARS is a useful predictor of reward sensitivity, but is a relatively poor predictor of effort sensitivity. This mirrors the main result of this study, which showed that reward sensitivity was selectively affected in the patient group vs controls, while effort sensitivity was preserved. More broadly, these results are consistent with the suggestion in the literature that a feature of apathetic behaviour is reduced reward sensitivity (Adam et al., 2013; Muhammed et al., 2016; Rochat et al., 2013).

- 1. *Control Motor Analyses*

To ensure that the primary results reported in the main manuscript were not due to differences in force output between the groups, we conducted several analyses to ensure that force profiles did not differ between patients and controls.

2.2.1. Failure Rates

Participants were rewarded only on trials in which they successfully achieved the target effort level. To determine if failure rates differed as a function of Group or Effort, we compared failure rates in patients with a two-way ANOVA on the factors of Group (patients, controls) and Effort level (1-6). This analysis revealed a main effect of Effort, such that failure rates increased as a function of effort level (*p* < .001). Critically, however, neither the main effect of Group nor its interaction with Effort was significant (both *p* > .12), demonstrating that failure rates did not differ across Groups. We also checked whether groups differed in how willing they were engaged in effort levels beyond their capacity to perform (i.e., Effort Level 6, or 110% MVC). Importantly, there was no significant difference in this parameter across Groups (*t*(23) = 0.86).

*2.2.2.* Trial History

To determine if performance on one trial (i.e., failure or success to reach the required target force) influenced decisions on subsequent trials, we first determined the probability of a participant accepting a given offer in the trial immediately following failed versus successful attempts at gathering a reward. We then conducted a 2 x 2 ANOVA on the factors of Group (patients, controls) and Trial History (preceding trial failed vs successful). This analysis demonstrated no significant main effects or interactions – specifically, the probability of a patient accepting an offer was the same, regardless of whether they failed or succeeded on the preceding trial (Group, *F*(1, 23) = 0.16; Trial History, (*F*(1, 23) = 0.84; Group x Trial History, *F*(1, 23) = 1.26). This shows that the effect of trial history did not significantly differ between groups.

*2.2.3.* Patient Force Output Data

The following analyses were conducted in order to verify that the shifts in indifference points reported in the main text could not be attributable simply to changes in force output.

2.2.3.1. *Initial MVCs*

To recapitulate, the analyses reported in the main text showed that the MVC at the beginning of each session did not differ between Groups (*t*(23) = 0.16). In addition, we examined the effect of fatigue on motor performance by comparing changes in maximum force output over the course of the test, but this was no different between the two groups (*t*(23) = 0.98). Thus, the higher reward indifference points in patients was not simply due to a reduced capacity to exert force. In addition, the time-to-peak contraction did not differ between Groups (patients, 2.44 ± 0.10s vs controls, 2.79 ± 0.15s, *t*(23) = 1.98). This suggested that differences in indifference points between patients and controls could not simply be due to differences in motor strength at the beginning of each session.

2.2.3.2. *Time-on-task Analyses*

In order to verify that there were no changes in force output **during** the experiment, we compared motor output between the first and second halves of each session (90 trials per half). Importantly, there were no differences in maximal grip force between groups, or across the experiment (Group, *F*(1, 23) = 1.29; Session Half, *F*(1, 23) = 3.27; Group × Session Half, *F*(1, 23) = 2.97). Similarly, patients’ time-to-peak contraction did not differ over the course of the experiment as a function of Group (patients, *F*(1, 23) = 0.83; Session Half, *F*(1, 23) = 1.03; Group × Session Half, *F*(1, 23) = 3.14). In summary, these analyses show that there were no significant differences in patients’ motor output at the beginning of each experimental session, nor during the sessions themselves. This therefore excludes the possibility that any shifts of effort indifference points were due to changes in force output.

1. **Supplementary Discussion**
   1. *Neural mechanisms underlying the dissociation of effort and reward sensitivity*

Although effort and reward sensitivity are clearly related, they are distinguishable concepts (Chong & Husain, 2016). For individuals to determine whether it is worth embarking on a course of action, they must consider the reward on offer and the amount of effort involved in that action (Chong et al., 2017; Chong, Bonnelle, & Husain, 2016; Croxson, Walton, O'Reilly, Behrens, & Rushworth, 2009). It is well-established that individuals differ in their sensitivity to *rewards*, and that a key structure in mediating reward sensitivity is the ventral striatum (Hahn et al., 2011; Yacubian et al., 2007). Similarly, many current models of *effort*-based decision-making have found that the sensitivity to effort costs varies significantly across individuals, and involves the prefrontal cortex and its mesocortical connections (Salamone, Correa, Farrar, & Mingote, 2007; Schweimer & Hauber, 2006). In order to derive the net utility for embarking on a course of action, it is therefore necessary to take into account the raw reward and effort magnitudes of that action, each of which are differentially weighted depending on individuals’ sensitivity to each component.

This conceptual distinction is supported by neurophysiological data indicating that areas of the corticostriatal network differentially support reward-sensitive computations and their integration with effort costs. For example, dopamine release in the rat nucleus accumbens (ventral striatum) consistently reflects the magnitude of upcoming rewards, but less reliably predicts effort costs (Gan, Walton, & Phillips, 2010; Wanat, Kuhnen, & Phillips, 2010). Similarly, other studies have found that the majority of dopaminergic neurons in the macaque substantia nigra pars compacta (SNc) encode the value of upcoming rewards alone, with only a subset incorporating effort costs to derive the net subjective utility of the action (Pasquereau & Turner, 2013). These data suggest that midbrain dopamine release scales with the value of upcoming rewards, but not necessarily the net utility of the action required to obtain them.

In contrast, other areas seem to be differentially sensitive to effort costs. For example, inactivation of the basolateral amygdala results in a shift in preference away from exerting effort, while preserving the preference for larger rewards (Floresco & Ghods-Sharifi, 2007). Similar results have been found following lesions to the anterior cingulate cortex (Schweimer & Hauber, 2005; Walton, Bannerman, Alterescu, & Rushworth, 2003; Walton, Bannerman, & Rushworth, 2002). These data suggest that the prefrontal cortex, and the mesocortical dopaminergic fibres that directly project to it from the dopaminergic midbrain, are important in processing the effort involved in an action and integrating it with reward value to derive a subjective utility of that action for the individual (Walton, Rudebeck, Bannerman, & Rushworth, 2007).

This neural separation of costs and benefits is a precedent for the results reported in this manuscript. Here, we found a dissociation between effort and reward sensitivity following lesions to the pallidonigral complex, which represents the principal output nuclei of the basal ganglia. Given the importance of midbrain dopaminergic signalling to reward-based computations, the inability of reward-sensitive information to be relayed via these lesioned nuclei to thalamus and cortex could underlie the deficits in reward-sensitivity shown here. In contrast, the direct mesocortical pathway projecting from the dopaminergic midbrain to effort-sensitive areas in the prefrontal cortex are able to bypass the affected pallidonigral complex, in order to potentially drive the effort-based computations in our patient group.

- 1. *Methcathinone-induced parkinsonism vs idiopathic Parkinson’s disease*

The results of the present study offer an interesting counterpoint to recently conducted studies on patients with idiopathic Parkinson’s disease (PD) (Chong et al., 2015; Le Bouc et al., 2016; Schmidt et al., 2008). A recent study using the identical paradigm showed that effort indifference points were lower in patients with PD tested OFF medication relative to age-matched controls (Chong et al., 2015). This contrasts with the results of the present study, in which effort indifference points did not differ between patients with methcathinone-induced parkinsonism and their healthy counterparts.

These contrasting results are likely driven by the distinctive pathophysiological mechanisms underlying the two conditions. The cardinal feature of idiopathic PD is degeneration of midbrain dopaminergic neurons within the substantia nigra pars compacta (SNc), which subsequently leads to reduced dopamine synthesis, and subsequent dysfunction of the nigrostriatal pathway. The SNc is known to be important in motivating effort-based responses – for example, dopaminergic lesions of the SNc in rodents selectively impair effort-based responses (Drui et al., 2014). In addition, there is evidence implicating mesocorticolimbic dysfunction in the motivational deficits in PD (Thobois et al., 2010). Taking these two pathologies together, the reduced effort sensitivity in PD shown in previous studies is thought to be driven by degeneration of dopaminergic neurons within the SNc, subsequent reduction in dopamine synthesis, and dysfunction in the nigrostriatal and mesocorticolimbic pathways.

In contrast, dopamine synthesis is *preserved* in methcathinone-induced parkinsonism. The neurotoxic effects of manganese, which is produced in the manufacture of methcathinone, are particularly pronounced within the globus pallidus and substantia nigra pars reticulata (Olanow et al., 1996). Importantly, evidence from animal models of the disease, human radiotracer studies, and human autopsy data indicate that midbrain dopaminergic neurons and the nigrostriatal pathway are intact (Colosimo & Guidi, 2009; Perl & Olanow, 2007; Sanotsky et al., 2007; Selikhova et al., 2008; Shinotoh et al., 1997; Wolters et al., 1989). This suggests that the deficits in methcathinone-induced parkinsonism do not arise from the degeneration of nigrostriatal dopaminergic afferents, or the depletion of striatal dopamine (as is the case in idiopathic PD). Instead, the deficits are more likely due to changes in post-synaptic dopaminergic signalling, intrinsic to the caudate/putamen and/or its connections to the globus pallidus (Guilarte & Gonzales, 2015).

Given that dopamine synthesis is spared in methcathinone-induced parkinsonism, dopamine may therefore continue to exert an influence on effort-based computations through direct connections to areas of the prefrontal cortex that are known to be sensitive to effort (e.g., the anterior cingulate and other areas of the medial prefrontal cortex)(Floresco & Ghods-Sharifi, 2007; Walton et al., 2002). Preserved extra-striatal dopaminergic projections, such as the mesocortical pathway, may therefore continue to drive effort-sensitive computations, by projecting directly to these areas and bypassing the lesioned pallidonigral complex.

- 1. *Caveats*

Clearly, the interpretations offered above should be made with caution, given the complexities of the cortico-basal-ganglionic circuitry, and the relatively small size of this patient group. Future animal studies more specifically targeting the areas implicated in methcathinone-induced parkinsonism might assist to confirm the mechanistic interpretations offered here. In addition, comparison to other suitable patient groups might be useful to confirm the specificity of these effects to methcathinone-induced parkinsonism. Nevertheless, the present data indicate that reward and effort sensitivity are in principle dissociable, and that in our patient group is likely to be mediated by specific lesions to the pallidonigral complex.

*Supplementary Table 1*

Structural MRI findings of the seven patients with methcathinone-induced parkinsonism.

| Patient | Basal ganglia lesions on T1-weighted MRI | Other structural brain lesions |
| --- | --- | --- |
| 1 | Symmetrical increase in T1-weighted MR signal in the globus pallidus bilaterally, extending to the substantia nigra and red nuclei. No other basal ganglia nuclei affected | Nil |
| 2 | Symmetrical increase in T1-weighted MR signal in the globus pallidus bilaterally, extending to the substantia nigra and red nuclei. No other basal ganglia nuclei affected | Cerebellar peduncles and deep cerebellar nuclei (dentate and fastigial nuclei) |
| 3 | Symmetrical increase in T1-weighted MR signal in the globus pallidus bilaterally. No other basal ganglia nuclei affected | Nil |
| 4 | Nil | Nil |
| 5 | Nil | Nil |
| 6 | Nil | Nil |
| 7 | Nil | Nil |

**References**

Adam, R., Leff, A., Sinha, N., Turner, C., Bays, P., Draganski, B., & Husain, M. (2013). Dopamine reverses reward insensitivity in apathy following globus pallidus lesions. *Cortex, 49*(5), 1292-1303. doi:10.1016/j.cortex.2012.04.013

Chong, T. T.-J., Apps, M., Giehl, K., Sillence, A., Grima, L. L., & Husain, M. (2017). Neurocomputational mechanisms underlying subjective valuation of effort costs. *PLoS Biology, 15*(2), e1002598.

Chong, T. T.-J., Bonnelle, V., & Husain, M. (2016). Quantifying motivation with effort-based decision-making paradigms in health and disease. *Progress in Brain Research, 229*, 71-100.

Chong, T. T.-J., Bonnelle, V., Manohar, S., Veromann, K.-R., Muhammed, K., Tofaris, G. K., . . . Husain, M. (2015). Dopamine enhances willingness to exert effort for reward in Parkinson's disease. *Cortex, 69*, 40-46. doi:10.1016/j.cortex.2015.04.003

Chong, T. T.-J., & Husain, M. (2016). The role of dopamine in the pathophysiology and treatment of apathy. *Progress in Brain Research, 229*, 389-426.

Colosimo, C., & Guidi, M. (2009). Parkinsonism due to ephedrone neurotoxicity: A case report. *European Journal of Neurology, 16*, e114-115.

Croxson, P., Walton, M., O'Reilly, J., Behrens, T., & Rushworth, M. (2009). Effort-based cost-benefit valuation and the human brain. *Journal of Neuroscience, 29*(14), 4531-4541.

Drui, G., Carnicella, S., Carcenac, C., Favier, M., Bertrand, A., Boulet, S., & Savasta, M. (2014). Loss of dopaminergic nigrostriatal neurons accounts for the motivational and affective deficits in Parkinson's disease. *Molecular Psychiatry, 19*(3), 358-367.

Floresco, S. B., & Ghods-Sharifi, S. (2007). Amygdala-prefrontal cortical circuitry regulates effort-based decision making. *Cerebral Cortex, 17*(2), 251-260.

Gan, J., Walton, M., & Phillips, P. (2010). Dissociable cost and benefit encoding of future rewards by mesolimbic dopamine. *Nature Neuroscience, 13*, 25-27.

Guilarte, T., & Gonzales, K. (2015). Manganese-induced parkinsonism is not idiopathic Parkinson’s disease: Environmental and genetic evidence. *Toxicological Sciences, 146*(2), 204-212.

Hahn, T., Heinzel, S., Dresler, T., Plichta, M. M., Renner, T. J., Markulin, F., . . . Fallgatter, A. J. (2011). Association between reward‐related activation in the ventral striatum and trait reward sensitivity is moderated by dopamine transporter genotype. *Human Brain Mapping, 32*(10), 1557-1565.

Le Bouc, R., Rigoux, L., Schmidt, L., Degos, B., Welter, M. L., Vidailhet, M., . . . Pessiglione, M. (2016). Computational dissection of dopamine motor and motivational functions in humans. *Journal of Neuroscience Methods, 36*(25), 6623-6633. doi:10.1523/JNEUROSCI.3078-15.2016

Muhammed, K., Manohar, S., Yehuda, M. B., Chong, T. T.-J., Tofaris, G., Lennox, G., . . . Husain, M. (2016). Reward sensitivity deficits modulated by dopamine are associated with apathy in Parkinson’s disease. *Brain*, aww188.

Olanow, C. W., Good, P. F., Shinotoh, H., Hewitt, K. A., Vingerhoets, F., Snow, B. J., . . . Perl, D. P. (1996). Manganese intoxication in the rhesus monkey A clinical, imaging, pathologic, and biochemical study. *Neurology, 46*(2), 492-498.

Pasquereau, B., & Turner, R. (2013). Limited encoding of effort by dopamine neurons in a cost-benefit trade-off task. *Journal of Neuroscience, 33*(19), 8288-8300.

Perl, D., & Olanow, C. (2007). The neuropathology of manganese-induced Parkinsonism. *Journal of Neuropathology and Experimental Neurology, 66*(8), 675-682.

Poniatowska, R., Lusawa, M., Skierczynska, A., Makowicz, G., Habrat, B., & Sienkiewicz-Jarosz, H. (2014). MRI brain findings in ephedrone encephalopathy associated with manganese abuse: Single-center perspective. *Polish Journal of Radiology, 79*, 150-155.

Rochat, L., Van der Linden, M., Renaud, O., Epiney, J. B., Michel, P., Sztajzel, R., . . . Annoni, J. M. (2013). Poor reward sensitivity and apathy after stroke: Implication of basal ganglia. *Neurology & Clinical Neurophysiology, 81*(19), 1674-1680.

Rodier, J. (1955). Manganese poisoning in Moroccan miners. *British Journal of Industrial Medicine, 12*(1), 21-35.

Salamone, J. D., Correa, M., Farrar, A., & Mingote, S. M. (2007). Effort-related functions of nucleus accumbens dopamine and associated forebrain circuits. *Psychopharmacology, 191*(3), 461-482.

Sanotsky, Y., Lesyk, R., Fedoryshyn, L., Komnatska, I., Matviyenko, Y., & Fahn, S. (2007). Manganic encephalopathy due to “ephedrone” abuse. *Movement Disorders, 22*(1337-1343).

Schmidt, L., d’Arc, B. F., Lafargue, G., Galanaud, D., Czernecki, V., Grabli, D., . . . Pessiglione, M. (2008). Disconnecting force from money: effects of basal ganglia damage on incentive motivation. *Brain, 131*(5), 1303-1310.

Schweimer, J., & Hauber, W. (2005). Involvement of the rat anterior cingulate cortex in control of instrumental responses guided by reward expectancy. *Learning and Memory, 12*, 334-342.

Schweimer, J., & Hauber, W. (2006). Dopamine D1 receptors in the anterior cingulate cortex regulate effort-based decision making. *Learning and Memory, 13*, 777-782.

Selikhova, M., Fedoryshyn, L., Matviyenko, Y., Komnatska, I., Kyrylchuk, M., Krolicki, L., . . . Sanotsky, Y. (2008). Parkinsonism and dystonia caused by illicit use of Ephedrone — A longitudinal study. *Movement Disorders, 23*, 2224-2231.

Selikhova, M., Tripoliti, E., Fedoryshyn, L., Matvienko, Y., Stanetska, H., Boychuk, M., . . . Sanotsky, Y. (2016). Analysis of a distinct speech disorder seen in chronic manganese toxicity following ephedrone abuse. *Clinical Neurology and Neurosurgery, 147*, 71-77.

Shinotoh, H., Snow, B., Chu, N., Huang, C., Lu, C., Lee, C., . . . Calne, D. (1997). Presynaptic and postsynaptic striatal dopaminergic function in patients with manganese intoxication: a positron emission tomography study. *Neurology, 48*, 1053-1056.

Thobois, S., Ardouin, C., Lhommée, E., Klinger, H., Lagrange, C., Xie, J., . . . Juphard, A. (2010). Non-motor dopamine withdrawal syndrome after surgery for Parkinson’s disease: predictors and underlying mesolimbic denervation. *Brain, 133*(4), 1111-1127.

Walton, M., Bannerman, D., Alterescu, K., & Rushworth, M. (2003). Functional specialization within medial frontal cortex of the anterior cingulate for evaluating effort-related decisions. *Journal of Neuroscience, 23*, 6475-6647.

Walton, M., Bannerman, D., & Rushworth, M. (2002). The role of rat medial frontal cortex in effort-based decision making. *Journal of Neuroscience, 22*(24), 10996-11003.

Walton, M., Rudebeck, P., Bannerman, D., & Rushworth, M. (2007). Calculating the cost of acting in frontal cortex. *Annals of the New York Academy of Sciences, 1104*(1), 340-356. doi:10.1196/annals.1390.009

Wanat, M., Kuhnen, C., & Phillips, P. (2010). Delays conferred by escalating costs modulate dopamine release to rewards but not their predictors. *Journal of Neuroscience, 30*, 12020-12027.

Wolters, E., Huang, C., Clark, C., Peppard, R., Okada, J., Chu, N., . . . Calne, D. (1989). Positron emission tomography in manganese intoxication. *Annals of Neurology, 26*, 647-651.

Yacubian, J., Sommer, T., Schroeder, K., Gläscher, J., Kalisch, R., Leuenberger, B., . . . Büchel, C. (2007). Gene–gene interaction associated with neural reward sensitivity. *Proceedings of the National Academy of Sciences of the United States of America, 104*(19), 8125-8130.
